# Commentary: Safety and efficacy of a novel fecal microbiota transplantation method using hydrogen nanobubble water without antibiotics or bowel cleansing in children with autism spectrum disorder

**DOI:** 10.3389/fped.2026.1838225

**Published:** 2026-06-12

**Authors:** David P. Ruttenberg

**Affiliations:** UCL Institute of Education, University College London, London, United Kingdom

**Keywords:** autism spectrum disorder, fecal microbiota transplantation, gut-brain axis, methodological validity, randomized controlled trial, regression to the mean

## Introduction

The gut–brain axis has emerged as a compelling frontier in autism spectrum disorder (ASD) research, and interest in fecal microbiota transplantation (FMT) as a therapeutic strategy has grown accordingly (1, 2). Against this backdrop, Shirotani et al. (3) report a novel FMT approach using hydrogen nanobubble water that eliminates antibiotic pretreatment and bowel preparation—a meaningful clinical advance if confirmed. The 29% reduction in Social Responsiveness Scale-2 (SRS-2) total scores over 30 weeks, observed across social communication, restricted behavior, and sensory subscales, is a signal worth taking seriously. This commentary aims to situate those findings within their methodological constraints and to outline what a rigorous confirmatory study would require.

## The absence of a control condition

The study's central limitation is its single-arm design. Without a control arm, observed improvements cannot be attributed specifically to SHIN-1, the Good Manufacturing Practice (GMP)-grade fecal microbial solution used in the trial, rather than to the natural developmental trajectory of children aged 5–12 over a 30-week period, to enhanced parental attention during trial participation, or to regression to the mean.

Regression to the mean is a particular concern here. At baseline, 86.7% of participants were classified as severe on the SRS-2—consistent with selective enrollment of children at a symptomatic peak. In such samples, subsequent scores tend to move toward the population mean regardless of any intervention, a phenomenon that is well characterised in the methodological literature (4). The authors do not acknowledge this effect in their limitations section, and no analysis is presented that would allow estimation of its potential contribution.

## Effect size in context

The 29% reduction observed here is a notable signal. Meta-analytic work on early autism interventions has documented that open-label, parent-rated study designs substantially overestimate effect sizes compared to blinded, controlled designs (5). This does not mean the observed effect is entirely artifactual, but it does mean the uncontrolled design prevents determination of how much of the observed change reflects genuine treatment response vs. expectancy effects and regression to the mean. The signal is large enough to justify a properly designed follow-up—and the authors are right to call for one.

## Primary outcome validity and the Gazefinder corroboration

The SRS-2 is completed by parents who are unblinded to treatment, creating potential for expectancy bias. The authors address this by correlating SRS-2 Social Communication and Interaction (SCI) scores with Gazefinder, a proprietary eye-tracking system. This analysis confirms that the SRS-2 measures something coherent at a given timepoint, consistent with prior validation studies (6, 7). The SRS-2 is a psychometrically sound instrument with well-established reliability and validity for cross-sectional ASD severity measurement. What this cross-sectional correlation cannot establish is whether changes in SRS-2 scores over time reflect genuine neurobiological change rather than shifted parental perception.

Longitudinal Gazefinder data collected at each assessment timepoint—demonstrating parallel improvements in objective gaze metrics—would have provided that validation. Its absence leaves the temporal validity of the primary outcome uncertain.

## Multiple comparisons and secondary outcomes

The study tests at minimum fifteen distinct outcomes across SRS-2 subscales, the Short Sensory Profile (SSP) total and subscales, the Gastrointestinal Symptom Rating Scale (GSRS), the Bristol Stool Form Scale (BSFS), the Patient Health Questionnaire-4 items (PHQ-4), the Patient Health Questionnaire-2 (PHQ-2), and the Generalized Anxiety Disorder-2 (GAD-2), with no correction for multiple comparisons and no pre-specified endpoint hierarchy. All secondary outcome findings should therefore be treated as exploratory and hypothesis-generating.

An additional concern attaches to the PHQ-4. This instrument was developed and validated in the adult general population (8). Its use in children aged 5–12 with ASD—many of whom have limited introspective language access and variable emotional expression—is not established and requires explicit justification and age-appropriate validation before reported improvements in anxiety and depressive symptoms can be meaningfully interpreted.

## Subgroup analysis and sample size

The differential response between children with (*n* = 22, 24% improvement) and without (*n* = 8, 45% improvement) gastrointestinal disorders is an interesting secondary observation. With eight participants in the non-GI subgroup, however, the estimate is too imprecise to support strong interpretation. This finding warrants dedicated investigation in a larger, adequately powered study rather than definitive claims at this stage.

## A factual inconsistency in the abstract

The abstract states that the 29% SRS-2 improvement was 'sustained at one year.’ No twelve-month follow-up data are presented anywhere in the manuscript, and no methods section describes a one-year assessment. This is an inconsistency between the abstract and the reported study content that should be corrected before publication.

## The path forward

The authors have introduced a genuinely novel FMT preparation method that, if validated, would meaningfully reduce procedural burden in pediatric populations. The mechanistic hypothesis—that hydrogen nanobubble water supports obligate anaerobic colonisation while suppressing pathogenic species, thereby modulating the gut–brain axis—is biologically coherent and testable.

What is required now is a randomised, placebo-controlled trial incorporating: (1) a sham enema control arm with identical procedural contact; (2) blinded or objective primary outcome measurement; (3) a pre-specified endpoint hierarchy with appropriate correction for multiple comparisons; (4) adequate sample size to support primary and subgroup analyses; (5) longitudinal objective biomarkers including repeated Gazefinder assessments and group-level metagenomics; and (6) confirmed independence of data analysis from the funding entity. A rigorous trial designed along these lines would allow the field to determine whether the promising signal observed in SHIN-1 translates into a reproducible and clinically meaningful treatment effect.
